# Evolution of environmental chemistry study program curricula in tertiary education: a case study and general implications

**DOI:** 10.1007/s11356-024-33756-2

**Published:** 2024-06-01

**Authors:** Peter Šebej, Jakub Urík

**Affiliations:** RECETOX, Faculty of Science, Masaryk University, Kamenice 5, 625 00 Brno, Czech Republic

**Keywords:** Environmental chemistry, University education, Curriculum, Feedback, Environment, Health

## Abstract

Societal and scientific progress has led to the formation of new study programs, often with multidisciplinary curricula. Guarantors and teachers of such programs must be prepared to quickly adapt to the needs and demands of students, society, the job market, and the commercial sphere since many issues start surfacing during the first years of the programs’ life. Here we share our experience with such a process in the study program “Environment and Health” taught since 2019 at Masaryk University in Brno, Czech Republic. Feedback from students and alumni allows for improvement of the curriculum and organization of the program. We show feedback loops from three perspectives: Feedback from immediate short-term experience can be incorporated within a year, medium-term feedback loops can manifest after several years, and long-term ones even decades. While current students usually perceive only short- or medium-term issues, the philosophy and structure of the program must be built by predicting societal and commercial needs in the following decades. Such long-term aspects are often counterintuitive to students’ vision, but still have to be considered for the program to remain attractive to new applicants. Balancing the original vision, preparing and applying changes, and dealing with feedback on all levels are key managerial challenges of successful study programs.

**Graphical Abstract:**

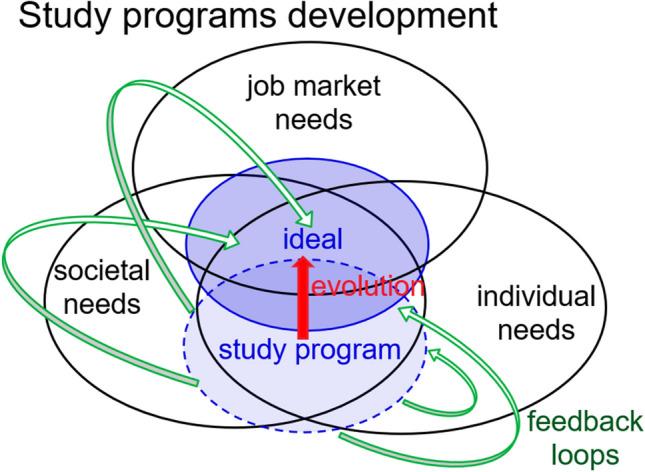

**Supplementary Information:**

The online version contains supplementary material available at 10.1007/s11356-024-33756-2.

## Introduction

Tertiary education is (typically) organized in large institutions (i.e., universities, colleges, professional and other schools, and here we will term all of them under a generic *university*) that are also actively engaged in research and other societal activities such as outreach, applied and/or for-profit development, consulting, and policy development.

At contemporary institutions, the students usually choose one study program (SP) that offers a multitude of courses, constituting a curriculum with pre-defined learning and training outcomes and an alumnus profile. The particular courses follow a course syllabus. An accreditation body typically assures and controls the quality of education and decides on the accreditation of SPs and/or institutions. Advances in knowledge, scientific discoveries, and societal changes have usually been quickly transferred into tertiary education, leading to pressure on changes in curricula. Quantitative and semi-quantitative comparison of SPs across European universities is simple using The European Credit Transfer and Accumulation System (ECTS)—a tool of the European Higher Education Area proposed by the Bologna declaration in 1999 (European Commission and Directorate-General for Education 2015).

### Creation and design of new study programs

In many fields, SPs and their curricula seem to be relatively stable and do not change quickly. However, even in such fields, we can historically trace changes and creations of new SPs, often by branching existing ones, in the same way as we can trace the “creation” of new scientific fields. This process often follows the lifecycle of the scientific paradigm shift, as described by Kuhn (Kuhn and Hacking 2012). A good example could be sprung out in the chemical disciplines. Organic chemistry stemmed from (general) chemistry. Biochemistry stemmed from organic chemistry. Others resulted from interactions of two or more fields, e.g., molecular biology arose where chemistry was necessary to understand biological processes. Environmental chemistry arose where physical, organic, and other chemical disciplines were necessary to understand processes in nature, and analytical science became a tool. In both these scenarios leading to new SPs, their creation and curation require strategic planning, with considerations of the skills and knowledge of future alumni and their participation in the job market in the long term. For programs arising at interfaces of fields, building and balancing the interdisciplinarity of SPs are also very important.

To simplify the dynamics of SPs, we could divide the relevant processes into (1) the formation of SPs; and (2) changes, evolution, and interactions during the program life cycle. These processes might span decades or more. At European universities, more specialized SPs (including at the bachelor level) are typical; however, at US universities, rather general SPs prevail. Discussions about the scope, depth, syllabi, and details of a new SP are especially crucial for the more specialized SPs, as they possess a higher risk of increased dropout ratio, fueled by students who realize the particular specialization does not suit them only during the studies.

### Why do we need feedback?

To keep potential students interested, actual students motivated, and alumni relevant for the job market, the curation of the SP curriculum is important. Inputs for curriculum management could come from feedback from all these groups, as well as from faculties, university management, and other stakeholders too (Benneworth and Jongbloed 2010; Jain et al. 2022). This idea is parallel to the fact that the success of for-profit companies depends on having working closed feedback loops, and such a strategy was also suggested to be implemented in governance (Whittle 2016). However, students’ feedback is primarily discussed within the context of a particular course or study unit (Malecka et al. 2022), but not so much in the context of SPs—higher hierarchy study units.

In this paper, we would like to shed light particularly on the evolution of SPs and feedback loops involved in this process and comment on the evolution of curricula of the study program Environment and Health (EH) at our institution as a case study and an example that allows us to suggest some generalizations. Our point of view is thus focused on programs in the sciences, especially those related to life, health, and environmental sciences.

## Discussion

### Emerging study programs and environmental chemistry

Many tertiary education SPs have names that give a very clear idea about the curriculum content to the general audience, e.g., biology, chemistry, and mathematics, and many others, especially at technical, trade, or other specialized institutions, also imply a clear connection to a particular job, e.g., chemical engineering or medical genetics. With the exception of programs with a connection to a professional career pathway, these are almost always representations of *traditional* and *established* fields. On the other hand, many of the emerging or multidisciplinary curricula, despite being created with very good reasoning, job-market research, and SWOT analysis, could run into issues of communication, misleading names, general misunderstandings, or even miss the potential auditoria (prospective students, employees, etc.).

The origins of SPs focused on environmental chemistry trace back to research on the observable effects of pollution on humans (e.g., “The great London smog”) (Stone 2002) or ecosystems (e.g., of DDT on populations of eagles) (Carson 1962). These have often been very closely related to analytical and physical chemistry but have been expanded to other subdomains of chemistry. In a recent study, it was shown that there are inconsistencies, and significantly variable shares of time/credits are now allocated to courses covering various chemical disciplines (Lammel et al. 2014). At about the same time, the formation of SPs in toxicology, ecotoxicology, and other fields relevant to the environment was advancing. Over time, the relationship between chemicals, environment, and health became the key concept in environmental and related fields, and the concept of the exposome with all its roles is now discussed in detail (Gao 2021; Barouki et al. 2022; Price et al. 2022). The concept of One Health similarly interconnects human, animal, and environmental health (Schwabe 1984; Zinsstag et al. 2011; Destoumieux-Garzón et al. 2018; Gao 2021). The emergence of such interdisciplinary concepts, research topics, approaches, paradigms, and changes in the philosophy of particular fields is often followed by the development of the SPs. In environmental sciences, this has led to the appearance of integrative programs covering a wide range of topics in environmental sciences and health, including the one at Masaryk University.

### Study program Environment and Health

Our SP Environment and Health (EH)—bachelor (*a*) and following master (*b*)—were created with the philosophy of education in the fields related and important for understanding environment and health, which translated into key requirements of:

- Solid chemical and biological background to build upon (mainly *a*)
- Orientation in environmental and (eco)toxicological issues (basics in *a*, in-depth in *b*)
- Interdisciplinary understanding and ability to interconnect knowledge (mainly *b*)
- Understanding and hands-on experience in a wide array of laboratory and field skills (both *a* and *b*)
- Presentation, teamwork, and other soft (transferable) skills (both *a* and *b*)

These programs started at Masaryk University in 2019 at the bachelor (3 years) and master (2 years) levels (see Supporting Information for details on organization of the tertiary education in the Czech Republic) and have been open for applications every year since. Typically, each year there are about 100–120 applicants and about 30 students starting the first year of bachelor SP.

### Feedback loops

To learn more about students’ views on the programs and expectations and to get inputs for data-based decisions on program management, we are continuously investigating the motivation of students, their understanding of the SP, their views, and opinions, by questionnaires, interviews, offering counseling by designated faculty members from the Department, and specialized counselors or ombudspersons at the level of school or university. Additional feedback is facilitated by an SP board (see Supporting Information for more details). Across the entire university, students evaluate courses and teachers using an anonymous questionnaire at the end of each term. Altogether, these represent a wealth of pathways for getting feedback from students on various aspects of courses, curricula, etc.

**Fig. 1:**
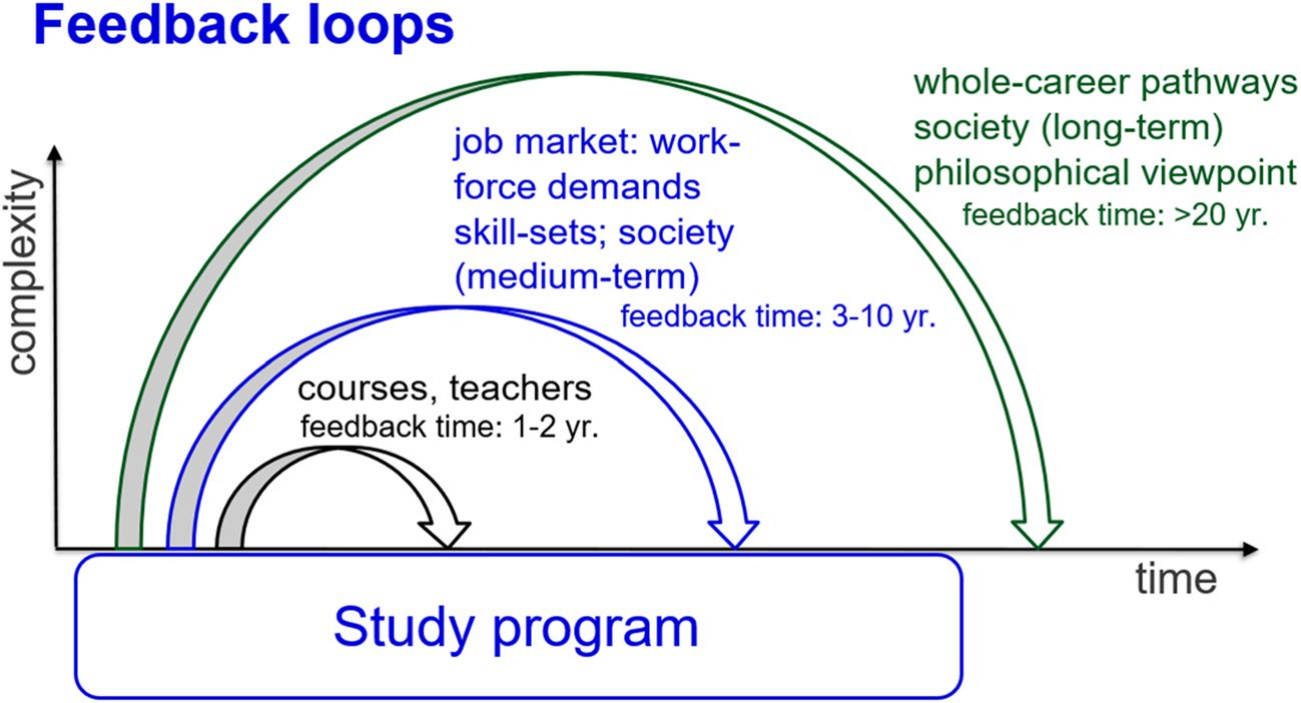
Lifetime of the study programs and a simplified formal diagram of feedback loops of different timescales

We divided the feedback into three roughly defined feedback loops: (a) short-term, (b) medium-term, and (c) long-term (Fig. 1). The division is based on the feedback’s nature and content; potential implementation of changes, suggested responses, and actions; and the time necessary for implementation and observation of the effects. The simplest questions and suggestions are often regarding the quality of in-course teaching, course syllabi, course integration in the curriculum (incl., e.g., mandatory/voluntary course status, substantial changes in syllabus), and addition of a new course or a course withdrawal, which all could be gathered, processed, and analyzed very quickly. In many cases, the changes based on the feedback analysis could be implemented within the next academic year (i.e., typical reaction and implementation time is 1–2 years) and are considered here as short term. Medium feedback loops involve, e.g., job market situation, increase in or even newly appeared demand for particular skill sets, emerging new jobs or jobs ceasing to exist, and societal changes, particularly short term. These are sometimes expressed, e.g., by large funding schemes (such as EU’s Framework Programmes for Research and Technological Development, with Horizon Europe running now) (European Commission 2023). Other important inputs are the recently launched European Skills Agenda for Sustainable Competitiveness, Social Fairness and Resilience (European Commission 2020) and the already set global goals of sustainable development (United Nations 2015). Lastly, the long-term loops involve analysis and predictions of whole-career pathways, long-term society changes such as the role and function of tertiary education and philosophy of education. From this perspective, the whole concept of education looks very different, and this perspective is not the focus of this work.

During the 5 years of existence of our SP Environment and Health (both bachelor and master), while collecting feedback in all the abovementioned ways, we often heard students sharing the same view, paraphrased: “I never thought, that there will be so much chemistry in the environmental sciences.” On the contrary, professionals in research and development acknowledge the relevance of the concept of “Chemistry as a central science” (Balaban and Klein 2006; Brown et al. 2022) in environmental sciences. The understanding of the need for such a profound chemical background in the curriculum was also reported by our alumni. However, a share of students reported that they were interpreting the name of the SP as one focusing on environmentalism, not environmental chemistry, and on health outcomes of environmental issues, not mechanisms and risks. This was answered with several responses. The quick response was the introduction of a 2-h, full-term course (2 ETCS) on chemical calculations (with a dedicated instructor and small groups of students, not more than 20) and several seminars explaining the concepts of the SP, exposome, and overview of interrelationships of environment, health, and necessary basic fields and changes in communication of the program to potential students. The space devoted to inorganic chemistry was reported by students to be unnecessarily high, as the original concept contained courses evenly covering all basic chemical disciplines. We significantly cut it down and the freed space was reallocated to chemistry calculations—a technical transferable skill itself (all within the first year of study). The medium-term response was the introduction of two specializations—(a) environmental chemistry and toxicology and (b) environmental health, starting in 2023. These immediate simple responses, such as the introduction of the chemical calculations course, had a very quick effect of decreasing the dropout rate from the study (from ca. 40% before to ~ 20% after) and decreasing the rate of students leaving for another SP.

Students who advanced into later years of study and alumni generally provide positive feedback about practical courses (laboratory and field) in the curriculum. These are relatively evenly distributed throughout all years of EH SP in the bachelor level and first year of the master level, which is generally appreciated by students, as it helps keep their motivation high by offering a chance to acquire hands-on practical experience throughout the study. However, we observed students asking for courses more closely related to health and environment, as the first 2 years of the curriculum are mainly introductory to many basic biology and chemistry topics, including practical courses (see Supporting Information for details). To follow this demand and this motivation channel, a field course in the first year, as well as an expanded time for a general introductory seminar, was added to the curriculum. In the master’s SP, the situation is different; the practical and hands-on courses are already specialized, and a fair share of them is based on a problem-solving approach (Jansson et al. 2015) and is well accepted by students.

**Fig. 2:**
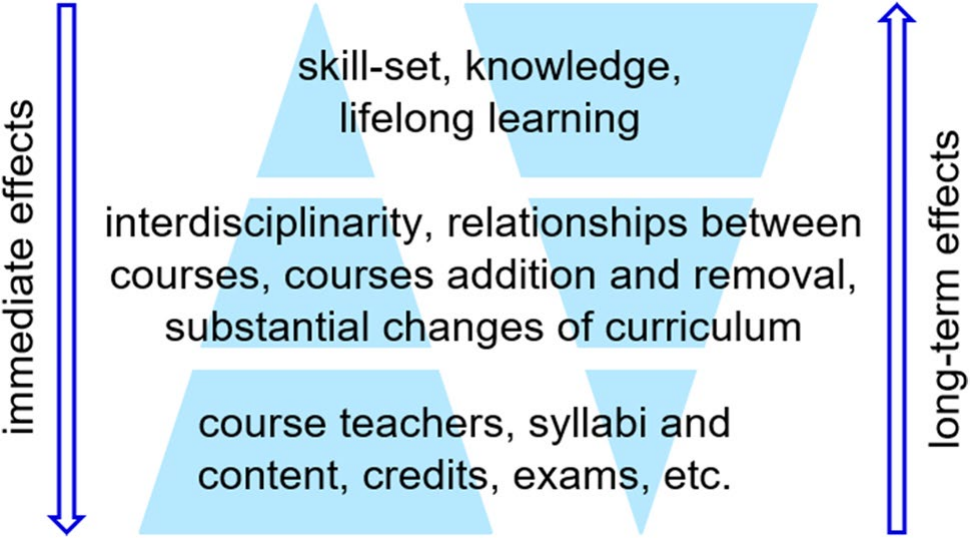
Feedback as perceived by students and from a long-term perspective

In line with the rapidly changing job market, emerging new skill sets and vanishing interest in others, and along a perspective of preparing alumni who will be pursuing their professional careers for about the next four decades, we put an emphasis on soft- and transferrable skills and bolster the will for and confidence in life-long learning as the key concept. To create space for their roles in the job market—both as a participant and as a creator—a series of mandatory seminars on transferrable skills (during the first 2 years of bachelor SP; 1 ETCS per term) was created and tailored to the anticipated needs of the EH SP. They cover areas such as (a) basic IT/software tools and knowledge, (b) basic scholarly skills, (c) scientific thinking, methods, and approaches to problems, and (d) presentation skills. On the basis of anonymous course feedback questionnaires and interviews with students, we found that they typically assess the courses and/or particular topics in the curricula through the lens of usefulness, especially in the short-term horizon (Fig. 2). We thought that this would lead, e.g., to understanding the usefulness of classes on skills such as primary and scientific literature search and writing. On the other hand, students often find the scientific thinking and research skills, shaping and falsifying hypotheses, a bit too little connected to their ongoing study, and perhaps welcome more general critical thinking. Thus, to paraphrase the students’ feedback (both anonymous and non-anonymous), teaching basic office software is useless because they know it well; scientific thinking is useless because they do not see a direct practical application; and presentation skills are very useful and should be allocated more hours. As a reaction, we substantially changed over half of the soft-skill seminar curriculum in the first term, decreased the timeshare allocated for general and office software packages, and allocated much more time for an introduction to specialized software tools (such as advanced graphs, basic data analysis, basic statistics, geographic information systems, infographics, biological and chemical objects, structures). However, we decided not to remove unpopular topics such as scientific thinking and instead focused on improving their attractiveness and communicating their usefulness.

### Long-term outlook

**Fig. 3:**
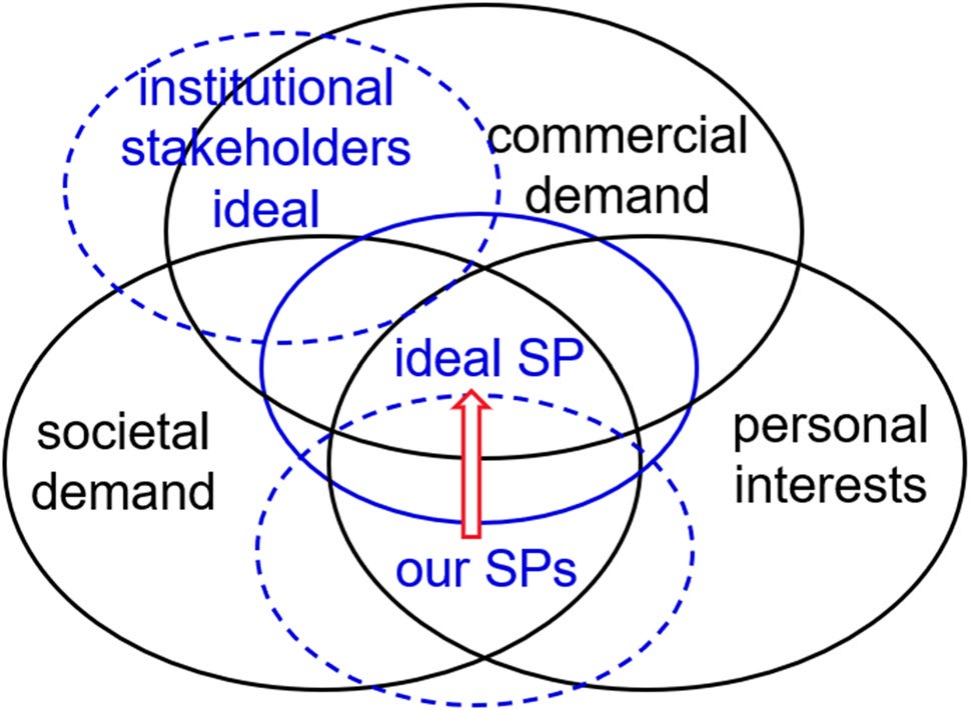
Conceptual viewpoint on a study program as a process of improvement. Stakeholders of higher education usually have different views on the ideal SP and only one (an example) is shown for clarity. Personal interests may include anyone related to the SP, e.g., students, teachers, or administrators, and their interests do not fully overlap

The given examples of feedback and responses are illustrative of the fact that we view the SP(s) not as a permanent curriculum but rather as a process (Fig. 3). This is particularly important for emerging fields and fields undergoing rapid changes (such as environmental sciences and health). However, most of the students’ feedback (including the described examples) confronts, comments, or questions courses and topics with regard to short-term usefulness. The overall philosophy of the SP is considered very rarely. In addition, one of the very typical questions is “What will be my job?” The options start surfacing only during the last year of the bachelor SP or even during the following master’s program, which we found to be discouraging to students, and they often expect training for a job market from the first year.

This correlates well with the question of the role of education, as discussed by Liessmann (Liessmann 2008). Commercial and, unfortunately, often also societal demand for education is to be primarily training for a role (e.g., a job type), not for the life of alumnus and future of the society, or simply as a value in itself. Often one of the most important parameters of a program’s success is the number of alumni that managed to find a job “in their field.” In natural sciences, that usually means focusing on the quality of hard science and practical skills. Improving society by creating an intellectual elite capable of tackling future complex issues that combine scientific, social, philosophical, economic, or political aspects is not an easily quantifiable aim and thus practically not an aim at all. However, complex issues, such as those in the environment and health, need such a complex approach.

To wrap up, we see value in students’ feedback on the SP curriculum, courses, and teachers. From the point of view of SP management, it is one of the quickest types of feedback and allows testing of solutions to reported issues and creating short feedback loops. Feedback processes are often difficult to manage, and feedback literacy should be cultivated on both students’ and teachers’ sides (Carless and Winstone 2023). They are an important element of students’ learning (Clynes and Raftery 2008) and a transferrable skill by itself.

## Conclusions and future remarks

Here we describe the formation and evolution of the curricula of the study programs Environment and Health. The role of feedback of various types and origins in these processes underlines the view of higher education study programs and curricula as a process. New specializations are now introduced in the discussed study programs, and they are partially based on the breadth of short- and medium-term feedback we have been obtaining. We will keep receiving and actively seeking feedback to keep the program alive and up to date.

## Supplementary Information

Below is the link to the electronic supplementary material.

Supplementary file1 (DOCX 85 KB)
