# Supplementary material for: Evolution of environmental chemistry study program curricula in tertiary education: a case study and general implications
Source: Environ Sci Pollut Res Int. 2024 Jun 1;33(22):11051–6. doi: 10.1007/s11356-024-33756-2 (PMC13415670; doi:10.1007/s11356-024-33756-2)
Supplement: Supplementary file 1 — Supplementary file1 (DOCX 85 KB) [file 11356_2024_33756_MOESM1_ESM.docx]

Supporting Information for

Evolution of Environmental Chemistry Study Programs Curricula in Tertiary Education: A case study and General Implications

Peter Šebej,^a,*^ Jakub Urík,^a^

^a^ RECETOX, Faculty of Science, Masaryk University, Kamenice 5, 625 00, Brno, Czech Republic

**Content**

[Organization of third level education in the Czech Republic S2](#_Toc155274601)

[Organization of the study program Environment and Health at Masaryk University S2](#_Toc155274602)

[Collection of feedback S4](#_Toc155274603)

[References S4](#_Toc155274604)

# Organization of third level education in the Czech Republic

The post-secondary education in the Czech Republic is provided by higher professional schools, offering specialized education suited for certain professional careers, and by universities. There are public and private universities, as well as state-run police and military training academies. Study programs in the Czech language at public universities are free for first-time attendants, which often leads to high drop-out rates (typically by students, who “are trying to find a suitable field” and/or by those, who “are just trying to study” to keep the student status and benefit from it), especially in the first year.

University education takes from 2 to 6 years (with two terms per year), depending on the degree of studies: Bachelor's degree programs usually last 3 years, followed by Master's degree programs, typically 2 years. Several programs are exceptions, e.g. in law, pharmacy, performing arts, dentistry (5-years programs) and medicine (6-years programs). For admission to the first year of bachelor programs and long (5- and 6-years master programs) it is mandatory to pass the universal leaving qualification of four-year (or longer) secondary schools. In addition, many programs have entry exams.

The study programs at universities are using the standardized European Credit Transfer and Accumulation System (ECTS) for awarding and comparing academic credits, *i.e.*, the "volume of learning based on the defined learning outcomes and their associated workload" for higher education across the European Union and other collaborating European countries (European Commission and Directorate-General for Education 2015), and has a threshold of 60 ECTS points per academic year.

# Organization of the study program Environment and Health at Masaryk University

The study programs Environment and Health at the RECETOX Centre, Faculty of Science, Masaryk University in Brno, Czech Republic, started with first students in Sept. 2018 and replaced the programs in Environmental Chemistry and in Ecotoxicology running for several decades. They are offered in bachelor level (3 years, 180 ECTS credit points) and following master level (2 years, 120 ECTS). In addition, the Centre is also offering bachelor and master programs in Mathematical Biology (with the same formal organization). The doctoral study program Environmental Health Sciences (4 years) is offered as highest-level tertiary education program at the Centre.

The bachelor program has its 180 credit points (cp) distributed as follows (Figure S1a):

1. Basic courses in chemistry (50 cp) and biology (48 cp); including lab. courses and seminars (22 and 3 cp);
2. Environment&Health: Overview and integrative (8 cp) and Introductory (25 cp) courses and Field trip (4 cp);
3. Soft-skills and research seminars (8 cp), mathematics and statistics (10 cp);
4. Others: English (4 cp); Physical Education (2 cp);
5. Optional courses (11 cp), with a choose from faculty-wide offer;
6. Work on the Bachelor thesis (10 cp).

The time of contact education is divided about half/half between lectures and seminars, laboratory courses and other practical courses, such as field trip and individual work on thesis (Figure 1b).

a)


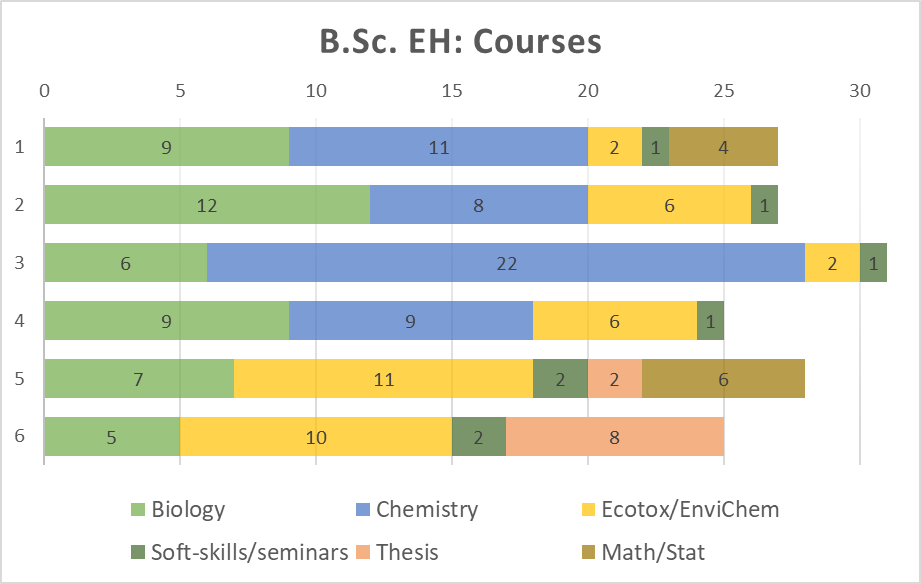


b)


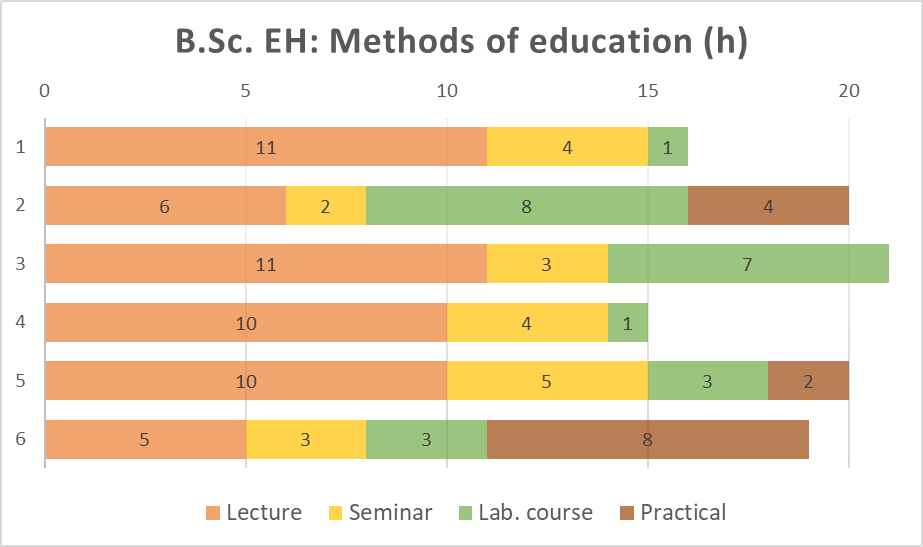


Figure S1. Anatomy of the bachelor study program Environment and Health (3 years), a) distribution of ECTS credit points by fields; and b) hours per week of particular type of education. Note: in both figures only mandatory courses are shown; the ECTS credits up to 180 per three years could be supplied from offer of free-to-choose courses across the RECETOX Department, Faculty of Science, or anywhere at the Masaryk University. The y-axis is terms (2 terms per academic year).

# Collection of feedback

Feedback from students was collected in several ways. At the end of each semester, students at Masaryk University are automatically given an anonymous survey to evaluate each course they attended. Questions tackle different aspects, e.g., educational value, clarity of lectures and teacher’s evaluation methods, quality of teacher’s preparation for the lectures, or how much time the student had to spend on the course. Students can also add any comments, positive or negative. The survey completion is voluntary, participation rates vary depending on faculty, teacher, and course, and is usually between 15% and 70%. In our program, we encourage the students to complete the survey, and especially provide written comments. However, the results are still rather unreliable for some courses due to the low rate of participation, or low number of participants.

Another means of providing feedback for students is through their representatives at the study program board. Each study program at Masaryk University on bachelor and master level has either its own board or shares board with similar programs. The board consists of guarantors, teachers, students, and at least one alumnus or representative of employers from outside the academy. Members meet in person at least annually, and their aim is to evaluate the quality of the program and propose and discuss changes. Study board of the Environment and Health study programs at MU consists of 15 members in total, including 7 student members, the rest are representatives of potential employees, alumni, faculty members and program guarantor(s).

Students of the Environment and Health program have one additional, more personal way of providing feedback. Each level has its own “study guide”, a lecturer dedicated to communication with students, advisor and contact for any study-related issues. The guide’s role is to build enough trust with students to allow direct reception of complaints, comments, and students’ views. There is also a similar role, an ombudsperson at the level of particular school or faculty.

Finally, at the beginning of their Bachelor’s degree studies, students receive a survey regarding their motivation for choosing our program as well as other questions about their interests and career views. This gives us a fair understanding of students’ expectations and views, both in general education and our study program.

# References

European Commission, Directorate-General for Education Y Sport and Culture (2015) ECTS users’ guide 2015. Publications Office of the European Union
